# Household Food Items Toxic to Dogs and Cats

**DOI:** 10.3389/fvets.2016.00026

**Published:** 2016-03-22

**Authors:** Cristina Cortinovis, Francesca Caloni

**Affiliations:** ^1^Department of Health, Animal Science and Food Safety, Universitá degli Studi di Milano, Milan, Italy

**Keywords:** chocolate, ethanol, grape, macadamia nuts, onion, poisoning, pets, xylitol

## Abstract

Several foods that are perfectly suitable for human consumption can be toxic to dogs and cats. Food-associated poisoning cases involving the accidental ingestion of chocolate and chocolate-based products, *Allium* spp. (onion, garlic, leek, and chives), macadamia nuts, *Vitis vinifera* fruits (grapes, raisins, sultanas, and currants), products sweetened with xylitol, alcoholic beverages, and unbaked bread dough have been reported worldwide in the last decade. The poisoning episodes are generally due to lack of public knowledge of the serious health threat to dogs and cats that can be posed by these products. The present review aims to outline the current knowledge of common food items frequently involved in the poisoning of small animals, particularly dogs, and provides an overview of poisoning episodes reported in the literature.

## Introduction

Several foods, while safe for humans, may pose a serious threat to the health of dogs and cats. Food-associated poisoning cases involving the accidental ingestion of chocolate, onions, macadamia nuts, *Vitis vinifera* fruits (grapes, raisins, sultanas, and currants), xylitol, and ethanol have been recorded worldwide in the last decade (1–3). Foods accounted for 14.8% of hazardous exposure cases reported to the Kansas State Veterinary Diagnostic Laboratory. Chocolate- and cocoa-based products were most commonly involved, followed by products sweetened with xylitol, onions and garlic, grapes and raisins, macadamia nuts, and ethanol (3). In general, the poisoning episodes resulted from a lack of public knowledge of the health hazard to small animals that may be posed by these products. Dogs and cats may be fed harmful foodstuffs by owners unaware of the danger or given the wide occurrence of these products in the home, pets may easily have accidental access to them. Dogs are undiscriminating in their eating habits and will readily ingest potentially harmful foodstuffs, thus being far more commonly affected than cats (4). While some foodstuffs, such as chocolate, have long been known to cause poisoning in dogs and cats, others such as grapes had previously been considered unlikely to cause problems and have emerged as a potential concern only in the last few years (5). As a consequence, cases of significant exposure had been wrongly diagnosed for many years (5). This review aims to outline the current knowledge of common household foods frequently involved in the poisoning of small animals, particularly dogs, and provides an overview of poisoning episodes reported in the literature.

## *Allium* spp.

Onion (*Allium cepa*), garlic (*Allium sativum*), leek (*Allium porrum*), and chives (*Allium schoenoprasum*) are all members of the genus *Allium* (Amaryllidaceae family). These bulbous plants are strongly aromatic, producing a characteristic odor when crushed, and are commonly used (fresh, cooked, or dehydrated) as ingredients in many dishes. The components responsible for their toxicity are organosulfoxides. Chewing the plant converts organosulfoxides to a complex mixture of sulfur compounds. The primary toxicological mechanism of *Allium*-derived sulfur compounds is oxidative hemolysis characterized by the development of methemoglobinemia and Heinz body formation in the erythrocytes (6, 7). Cooking, drying, and processing do not eliminate the toxic effect of *Allium* spp. (7, 8). Dogs and cats are highly susceptible to *Allium* toxicosis and the ingestion of 5 g/kg of onions by cats and 15–30 g/kg by dogs is enough to cause clinically important hematologic changes (8). In the case of dogs, hereditary high erythrocyte-reduced glutathione and potassium concentrations observed in certain breeds (e.g., Akita, Shiba, and Jindo) lead to greater susceptibility to onion-induced oxidative damage (9). Clinical signs of *Allium* toxicosis may appear 1 day or several days after consumption depending on the amounts ingested. Common clinical signs initially include vomiting, diarrhea, abdominal pain, loss of appetite, and depression. Due to the developing anemia, pale mucous membranes, weakness, rapid respiratory and heart rates, jaundice, and dark urine (reddish or brown) indicating hemoglobinuria are subsequently observed (7). Several cases of dog and cat poisoning by *Allium* spp. have been reported in the literature. Poisoning has been reported to occur after the ingestion of Catalan spring onion commonly known as “calcot” (10), baked garlic (11), onion soufflè (12), butter-cooked onions (13), and Chinese steamed dumplings containing Chinese chives (*Allium tuberosum*) and garlic (14). A case in which a dog was intentionally fed a large quantity of raw onions by the owner has also been reported (15). From 1994 to 2008, 69 cases of canine poisoning and 4 cases of feline poisoning by *Allium* spp. ingestion (16) were recorded by the Veterinary Poisons Information Service (VPIS). In the case of dogs, vomiting, diarrhea, abdominal pain and, less frequently, anemia, hematuria, and convulsions were reported. Two cases of death occurred and two dogs were euthanized. In the case of cats, gastrointestinal signs, lethargy, and polydipsia occurred in one case, while anemia and icterus were observed in the second case. One cat remained asymptomatic without treatment, and the other cat died from hemorrhage into the pleural and abdominal cavities (16). Recently, hypertension associated with garlic-induced hemolytic anemia has been reported in the case of a dog (11). No specific antidote is available for *Allium* toxicosis. Inducing vomiting should be considered in asymptomatic dogs and cats, provided there are no complicating factors and not more than 2 h have elapsed since ingestion (8). The administration of activated charcoal is indicated after vomiting has stopped. Once clinical signs have manifested themselves, treatment should consist of supportive care. Severely anemic animals may require a blood transfusion (8).

## Ethanol

Ethanol or ethyl alcohol is a two-carbon alcohol found in a variety of products, such as alcoholic beverages, paint and varnish, medication, perfume, mouthwash, certain types of thermometers, and certain forms of antifreeze. It is also used as a disinfectant, a fuel substitute, and if administered intravenously, as a competitive substrate in the treatment of dogs and cats poisoned by ethylene glycol. Ethanol toxicosis in small animals generally occurs as a result of accidental ingestion of alcoholic beverages (17–20). Ethanol intoxication has also been reported in the case of dogs, following ingestion of rotten apples (21), sloe berries used to make sloe gin (22), and uncooked bread and pizza dough (23–25). The latter contains the yeast *Saccharomyces cerevisiae*, which metabolizes carbohydrate substrates to ethanol and carbon dioxide (26). Once ingested, ethanol is rapidly absorbed from the gastrointestinal tract and crosses the blood–brain barrier (27). The mechanism of action of ethanol is not completely clear. Ethanol is suspected of inhibiting *N*-methyl-d-aspartate glutamate receptors in brain cells and the related production of cyclic guanosine monophosphate (27). Clinical signs usually develop within an hour of ingestion and include central nervous system (CNS) depression, ataxia, lethargy, sedation, hypothermia, and metabolic acidosis (27). Animals may become comatose and develop severe respiratory depression. A distended and painful abdomen due to excessive gas production may be noted in animals that ingest uncooked bread dough (25). Emesis should be induced with extreme caution and only in cases of very recent ingestion by animals that prove to be asymptomatic (27). Recently, hemodialysis has been shown to be beneficial for the rapid removal of ethanol in patients with severe toxicosis (20). Yohimbine, an alpha 2-adrenergic antagonist which readily crosses the blood–brain barrier, has been recommended as an arousal agent in the treatment of ethanol intoxication (25). In previous case reports, most patients recovered when monitored closely and given supportive care (22, 24, 28). Fatal ethanol intoxication was reported in the case of a dog, following the massive ingestion of rotten apples. The dog developed vomiting, ataxia, tremors, and dehydration, and died 48 h later. However, liver damage secondary to the chronic ingestion of rotten apples (presumed to reflect chronic ethanol toxicity) was suspected as a factor in the death of this dog (21).

## Grapes and Their Dried Products (Raisins, Sultanas, and Currants)

Grapes, the fruits of *Vitis vinifera*, and their dried products (raisins, sultanas, and currants) have been reported to cause renal failure in dogs. The fruits may be ingested raw or cooked as ingredients of fruit cake, mince pies, malt loaf, snack bars, scones, and other baked goods (28). The toxic syndrome has also been observed with consumption of marc (the residue of grapes after pressing) (29). The toxic principle(s) and the exact mechanism of grape-induced nephrotoxicity are still unknown. The latter appears to involve a nephrotoxic agent or an idiosyncratic reaction, leading to hypovolemic shock and renal ischemia (30). There is considerable variation in the susceptibility of dogs to grapes and their dried products. In a recent study that reviewed 180 reports recorded by the VPIS between August 1994 and September 2007 on the ingestion of *Vitis* fruits by dogs, some animals were reported to remain asymptomatic after ingesting up to 1 kg of raisins while others died following the ingestion of just a handful (28). Published case reports have identified renal failure in dogs following the ingestion of estimated doses of raisins as low as 2.8 mg/kg (31) and as little as four to five grapes in a dog weighing 8.2 kg (32) (Table 1). Therefore, ingestion of any quantity of these fruits should be considered as a potential clinical problem. Vomiting within 24 h of ingestion is the typical clinical sign observed. Diarrhea, anorexia, lethargy, and abdominal pain have also been reported (28, 31). Partially digested grapes and grape products may be present in the vomit, fecal material, or both (31–33). This is followed by signs of renal insufficiency or failure (oliguria, anuria, polydipsia, proteinuria, and elevated serum concentrations of creatinine and urea) within a short period (28, 31, 32). In cases of dogs with oliguria or anuria, the prognosis is generally poor (30, 34). The time taken to administer treatment may also play a significant role in the outcome (28). Given the large variability in the tolerance exhibited by dogs, the ingestion of any quantity of grapes or grape products by dogs should be handled aggressively (28). Following recent ingestion, prompt decontamination using emetics and repeated doses of activated charcoal is highly recommended (28, 30). All dogs should receive aggressive intravenous fluid therapy for a minimum of 48–72 h, and their renal function should be monitored for at least 72 h (28, 30).

**Table 1:** **Range of doses of grapes and their dried products (raisins, sultanas, and currants) reported to cause renal failure in dogs**.

Breed	Age (years)	Sex	Product	Dose	Time to onset of clinical signs (h)	Outcome	Reference
Labrador Retriever (13 dogs) Golden Retriever (4 dogs) Others (26 dogs)	Range: 0.6–13, median: 4	Male (27 dogs) Female (16 dogs)	Raisins (28 dogs) Grapes (13 dogs) Raisins and grapes (2 dogs)	Raisins^a^: range: 2.8–36.4 g/kg, median: 19.6 g/kg	<24 (35 dogs) 24–48 (8 dogs)	Recovery (23 dogs) Died (5 dogs) Euthanized (15 dogs)	(31)
				Grapes^b^: range: 19.6–148.4 g/kg, median: 40.6 g/kg			
Labrador Retriever	3	Male	Raisins	3 g/kg	–^c^	Died or euthanized	(35)
Dachshund	5	Male	Grapes	4–5 grapes in an 8.2-kg dog	–	Recovery	(32)
German Shepherd Dog cross	4	Male	Grapes	4 g/kg	–	Died or euthanized	(35)
Norwegian Elkhound	5	Male	Raisins	4.8 g/kg	–	Died	(32)
Labrador Retriever cross	3.5	Male	Grapes	6 g/kg	–	Died or euthanized	(35)
American Pit Bull Terrier	8.5	Male	Raisins	6 g/kg	–	Died or euthanized	(35)
Labrador Retriever	3.5	Male	Currants	7.8 g/kg	12	Recovery	(36)
–	–	–	Grapes (5 dogs)	11.6–31.2 g/kg^d^	Few hours	Recovery (5 dogs)	(33)
			Raisins (5 dogs)			Died (2 dogs)	
						Euthanized (3 dogs)	
Labrador Retriever	6	Female	Raisins	15.7 g/kg	8	Died	(32)
Australian Shepherd Dog	7	Male	Raisins	18 g/kg	–	Died or euthanized	(35)
Maltese	2	Female	Grape skins	18.8 g/kg	12	Recovery	(37)
Galgo Espaniol	1	Female	Grapes and marc	20 g/kg	6–7	Euthanized	(38)
Maltese	3	Male	Grapes	20 g/kg	5	Died	(39)
Border Collie	3.5	Male	Raisins	20.6 g/kg	12	Recovery	(32)
Basenji	10	Female	Raisins	24 g/kg	–	Died or euthanized	(35)
Golden Retriever	2	Male	Raisins	30 g/kg	–	Died or euthanized	(35)
Labrador Retriever	7	Male	Raisins	30 g/kg	–	Died or euthanized	(35)
Maltese	2	Female	Grapes	65.2 g/kg	8	Euthanized	(40)

*^a^Data are limited to 24 dogs*.

*^b^Data are limited to four dogs*.

*^c^Unknown or not provided*.

*^d^Data are limited to four dogs*.

## Hops

Hops, the inflorescences of the female plant of the species *Humulus lupulus*, are used for beer brewing to add a bitter taste and hoppy aroma to beer and to stabilize the beer foam. As home brewing becomes increasingly popular, companion animals may be at risk of exposure. The ingestion of both fresh and spent hops has been associated with the development of malignant hyperthermia in dogs (41). Any breed of dog may be affected, but breeds predisposed to malignant hyperthermia (e.g., Greyhounds, Labrador Retrievers, Saint Bernards, Pointers, Dobermans, Border Collies, English Springer Spaniels, and northern breeds) appear to be particularly susceptible (42). Hops contain a variety of compounds, including resins, essential oils (hycrocarbons and oxygenated compounds), phenolic compounds (tannins), and nitrogenous compounds (43). These compounds or their metabolites may uncouple oxidative phosphorylation resulting in malignant hyperthermia (6, 42). Clinical signs may be seen within hours of consuming hops and include marked hyperthermia, anxiety, tachycardia, tachypnea, panting, vomiting, abdominal pain, and seizures. Dark brown urine suggesting muscle necrosis may be observed (6, 42). Mortality can be high despite the aggressive therapy for hyperthermia (6). Duncan et al. (44) reported five cases involving dogs (four of which were Greyhounds) that exhibited marked hyperthermia, restlessness, panting, vomiting, signs of abdominal pain, and seizures, after the ingestion of hops. Four of the five dogs died despite intensive care treatment (44). Dogs suspected of recent ingestion of hops should be treated aggressively to decontaminate the gastrointestinal tract. Cooling measures, such as ice baths and cold IV fluids, should be used to lower the high body temperature. If available, dantrolene sodium, a skeletal muscle relaxant that has been used in humans to reverse malignant hyperthermia, may be administered (6, 41, 42).

## Macadamia Nuts

Macadamia nuts are produced by trees of the genus *Macadamia* (Proteaceae family). Only two species, *Macadamia integrifolia* and *Macadamia tetraphylla*, are plants of commercial importance as sources of food. All *Macadamia* spp. accumulate cyanogenic glycoside (proteacin and durrin) in their seeds but in very low concentrations in the case of commercial seeds (45). Macadamia nuts are very popular as snacks for human consumption, both as plain nuts or when used in cakes, cookies, or candy (46). They are considered to be a valuable food for their low cholesterol and sodium content and as an excellent source of manganese and thiamine (45). Macadamia nut toxicosis has only been reported in dogs to date (1, 47–49). The mechanism of action of their toxicity in the case of dogs is currently unknown and the dose required to induce toxicity has not been established precisely (46). The ingestion of as little as 0.7 g/kg of nuts has been associated with the development of clinical signs in dogs (48). In another case, a series of toxic doses ranging from 2.2 to 62.4 g/kg was reported (49). Clinical signs generally develop within 12 h of ingestion and may include weakness (particularly hind limb weakness), depression, vomiting, ataxia, tremors, hyperthermia, abdominal pain, lameness, stiffness, recumbency, and pale mucous membranes (47–49). Although macadamia nut poisoning is relatively infrequent, 83 cases were reported in Queensland, a major area for *Macadamia* spp. cultivation in Australia, over a period of 5 years (1). No deaths have been reported to date, and animals are expected to fully recover within 24–48 h with minimal veterinary intervention (46). The induction of emesis should be considered in cases of recent ingestion of macadamia nuts by dogs that prove to be asymptomatic (46).

## Methylxanthines (Caffeine, Theobromine, and Theophylline)

Methylxanthines, including caffeine, theobromine, and theophylline, are plant-derived alkaloids that are commonly found in a variety of foods, beverages, human medication, and other products in the home. Caffeine is found in coffee (from the fruit of *Coffea arabica*), tea (from the leaves of *Thea sinensis*), guarana (from the seeds of *Paullinia cupana*), and as an additive in many soft drinks. Theobromine occurs in cacao seeds (*Theobroma cacao*) and in products manufactured from these seeds, such as chocolate. Theophylline is found in tea along with caffeine. Moreover, caffeine is used in human medication to increase mental alertness, and theophylline is widely used as a bronchodilator in anti-asthma drugs (50). Methylxanthines antagonize cellular adenosine receptors and inhibit cellular phosphodiesterases, causing an increase in cyclic adenosine monophosphate (cAMP) (50). Moreover, methylxanthines enhance the release of catecholamines and increase cellular calcium entry while inhibiting intracellular sequestration of calcium by the sarcoplasmic reticulum, leading to increased muscular contractility (51). These combined actions result in the stimulation of both the CNS and cardiac muscle, the relaxation of smooth muscle, most notably bronchial muscle, and diuresis (50). Methylxanthine poisoning cases have been reported after the ingestion of herbal supplements containing guarana (52), garden mulch made of cacao bean shells (53, 54), caffeine tablets (55, 56), and caffeine-containing bait (57). However, most poisoning cases occur as a result of chocolate ingestion (4, 58–62). Though the intoxication of cats may also occur, dogs are most commonly affected because of their indiscriminate eating habits (4). The presence of chocolate was noted in the 10 most common cases of toxicosis involving dogs reported to the VPIS and to the American Society for the Prevention of Cruelty to Animals (ASPCA), Animal Poisons Control Center (APCC) in the past few years (60, 63). Poisoning episodes frequently occur around holidays when there is a higher occurrence of chocolate products in the home (4, 60). In addition to theobromine, chocolate contains caffeine but in much lower concentrations. Theobromine and caffeine concentrations vary according to the type of chocolate (4). Unsweetened baking chocolate and cocoa powder usually contain more than 14 mg of theobromine per gram. Semisweet dark chocolate and milk chocolate often contain around 5 and 2 mg of theobromine per gram, respectively, while white chocolate is considered to be an insignificant source of theobromine (50). According to the ASPCA APCC data, mild clinical signs appear in dogs after ingesting 20 mg/kg of theobromine and caffeine, while severe clinical signs are observed at 40–50 mg/kg and seizures occur at 60 mg/kg (4). Dogs with CYP1A2 deficiency polymorphism 1117C > T may be more at risk of poisoning due to reduced metabolism (64, 65). In dogs, caffeine is absorbed rapidly after ingestion while theobromine is absorbed 10 times slower, reaching peak plasma levels at approximately 10 h (50). Initial clinical signs are generally observed within 2–4 h after ingestion and include restlessness, polydipsia, urinary incontinence, vomiting, and perhaps diarrhea. Dogs can be in an excited state and show marked hyperthermia and tachycardia. As intoxication progresses, hypertension, cardiac arrhythmias, premature ventricular contractions, muscular rigidity, ataxia, seizures, and coma may be observed. Death may occur from cardiac arrhythmia or respiratory failure (50). Decontamination *via* emesis or gastric lavage, administration of multiple doses of activated charcoal, and meticulous supportive care should be the mainstay of treatment (4, 60). Prognosis is usually good, if effective decontamination is obtained within 2–4 h of ingestion (50, 60).

## Xylitol

Xylitol is a five-carbon sugar alcohol primarily used as an artificial sweetener in many products, including sugar-free gum, candy, bread, cookies, and other baked goods. It can also be purchased as a granulated powder for cooking and baking. Because of its antibacterial activity and palatability, xylitol is also included in a variety of medical and dental care products. An additional concern is that the use of xylitol is not just confined to products intended for human use. Xylitol is also an ingredient in drinking water additives developed to help maintain dog and cat dental health (66). The increased marketing and use of xylitol as a sweetener in recent years has led to increased risk of pet exposure to this agent (66, 67). Dogs are the species at the risk of developing severe, life-threatening clinical signs. In dogs, xylitol is a potent stimulator of insulin release, leading to dramatic decrease in blood glucose levels (66, 67). Doses, as low as 0.03 g/kg, have resulted in hypoglycemia in this species (68). Moreover, xylitol ingestion has been associated with liver failure in dogs. The mechanisms responsible for hepatic injury are not clear yet. It is thought to be related to either adenosine triphosphate (ATP) depletion secondary to xylitol metabolism, leading to hepatic necrosis or the generation of hepatocyte-damaging reactive oxygen species or both (69). According to the ASPCA APCC data, the lowest dose associated with xylitol-induced liver failure in dogs is 0.5 g/kg (70). Clinical signs of xylitol toxicity in dogs may be related to hypoglycemia or hepatopathy or both. Vomiting is usually the initial clinical sign. Clinical signs of hypoglycemia, including lethargy, ataxia, collapse, and seizures, may develop within 30–60 min after ingestion or may be delayed up to 12 h after ingestion (66, 70). In dogs developing hepatopathy, lethargy, icterus, vomiting, and coagulopathic signs, such as petechiae, ecchymoses, and gastrointestinal hemorrhages, may be observed (67, 69). Several cases of xylitol ingestion have been reported in the last decade (69, 71–75). Table 2 summarizes the cases of xylitol-induced acute hepatic failure reported in the literature. Recently, a retrospective study of the 192 cases of xylitol ingestion by dogs, reported to three American university teaching hospitals from December 2007 to February 2012, has been performed (68). The median estimated dose ingested was 0.32 g/kg (range 0.03–3.64 g/kg). Clinical signs developed in 41 (21%) dogs and primarily included vomiting and lethargy, followed by diarrhea, ataxia, seizures, restlessness, and anorexia (68). Thirty dogs (15.6%) became hypoglycemic and no significant difference was observed between hypoglycemic and euglycemic dogs with regards to the estimated dose of xylitol ingested. No dogs developed clinical signs or showed biochemistry values consistent with liver failure. All dogs survived and were discharged, suggesting an excellent prognosis for dogs that receive prompt veterinary care and evade liver failure (68). Supportive care and monitoring are the mainstay of xylitol toxicity treatment. The induction of emesis should only be attempted early and in asymptomatic animals. Activated charcoal is not recommended because of its poor ability to bind to xylitol (68, 70). Blood glucose levels and liver function should be monitored. If hypoglycemia develops, intravenous dextrose should be administered (69, 70). Xylitol ingestion should always be considered by veterinarians as a differential diagnosis for any unexplained hypoglycemic presentation with or without accompanying liver dysfunction (76).

**Table 2:** **Range of xylitol doses reported to cause acute hepatic failure in dogs**.

Breed	Age (years)	Sex	Product	Dose (g/kg)	Time to onset of clinical signs (h)	Outcome	Reference
Standard Poodle	3	Male	5 or 6 cookies	1.4–2	24	Died	(69)
English Springer Spaniel	2.5	Male	Homemade bread containing xylitol	3.7	1–2	Recovered	(72)
Labrador Retriever	8	Female	Xylitol powder	4.1	2	Recovered	(69)
Cocker Spaniel	4	Male	45 pieces of gum	4.7	72	Died	(73)
Dalmatian	6	Female	8 muffins sweetened with xylitol	5	9	Recovered	(69)
Scottish Terrier	5	Female	30 pieces of gum	7	24	Euthanized	(69)
Labrador Retriever mixed breed	6	Female	Xylitol powder	13.9	24	Died	(69)
Welsh Springer Spaniel	4	Male	4 large chocolate-frosted muffins sweetened with xylitol	15.9	1–2	Euthanized	(69)
Miniature Dachshund	7	Female	100 pieces of gum	16	72	Recovered	(69)
Chihuahua	9	Male	Granulated xylitol	45	1–2	Recovered	(75)

## Conclusion

The present review highlights the issue of exposure of small animals, particularly dogs, to potentially harmful foodstuffs commonly present in the home. A noticeable trend in exposure has emerged due to the increasing popularity of xylitol as a sweetener in several products. Obtaining an accurate history of exposure, early recognition of clinical signs, and rapid establishment of appropriate therapy can greatly improve the prognosis of food-related poisoning cases. Large gaps still exist in public knowledge of the hazard that certain foodstuffs may pose to the health of dogs and cats. Preventing exposure is the key to reducing the incidence of these poisoning episodes. Therefore, it is important to increase the knowledge of pet owners with regard to foodstuffs that must not be fed to dogs and cats and should be stored outside their reach.

## Author Contributions

CC and FC gave substantial contributions to the conception and design of the work; drafted the work and revised it critically for important intellectual content; gave final approval of the version to be published; and agreed to be accountable for all aspects of the work in ensuring that questions related to the accuracy or integrity of any part of the work are appropriately investigated and resolved.

## Conflict of Interest Statement

The authors declare that the research was conducted in the absence of any commercial or financial relationships that could be construed as a potential conflict of interest. The reviewer PZ and handling Editor declared their shared affiliation, and the handling Editor states that the process nevertheless met the standards of a fair and objective review.
